# Efficacy and safety of artemether-lumefantrine (AL) and artesunate-amodiaquine (ASAQ) for the treatment of uncomplicated *Plasmodium falciparum* malaria among children 6–59 months in three sentinel sites of Sierra Leone, 2021–2022

**DOI:** 10.1186/s12936-026-05850-y

**Published:** 2026-04-09

**Authors:** Irene Cavros, Francisca Abanyie, Anitta Renitta Yokoe Kamara, Fay Chalobah, Nelson Fofana, Jehan Ahmed, Kwabena Larbi, Culzean Kennedy, Breanna Horton, Marko Bajic, Mashanika Richardson, Bryce Matlock, Jessica McCaffery, Santigie Kanu, David Schnabel, Temitayo S. A. Labor, Shelby Cash, Mateusz Plucinski, James P. Komeh, Musa Sillah-Kanu

**Affiliations:** 1 Malaria Branch, U.S. Centers for Disease Control and Prevention, Atlanta, GA USA; 2 National Malaria Control Programme, Ministry of Health and Sanitation, Freetown, Sierra Leone; 3 U.S. President’s Malaria Initiative Impact Malaria Project, Population Services International, Freetown, Sierra Leone; 4 U.S. President’s Malaria Initiative Impact Malaria Project, Population Services International, Washington, DC USA; 5 Department of Pediatrics, Emory University School of Medicine, Atlanta, GA USA; 6 U.S. Centers for Disease Control and Prevention, Freetown, Sierra Leone; 7 Pharmacy Board of Sierra Leone, Ministry of Health, Freetown, Sierra Leone

**Keywords:** Malaria, Efficacy, Artemether-lumefantrine, Artesunate-amodiaquine, *Pfcrt*, *Pfmdr1*, *Pfk13*, Sierra Leone, Antimalarial resistance

## Abstract

Antimalarial drug resistance leads to increased treatment failures, prolonged illness, and increased mortality. The World Health Organization (WHO) recommends malaria-endemic countries regularly (ideally biennially) assess the efficacy of commonly used antimalarials. An evaluation of Sierra Leone’s first and second-line antimalarials assessed artemether-lumefantrine (AL) in three sentinel sites—Bo, Bombali, and Kenema—and artesunate-amodiaquine (ASAQ) in two sites—Bo and Bombali. Children 6 months to 5 years old with uncomplicated malaria were enrolled in the 28 day in vivo efficacy study using the WHO-recommended protocols. Genotyping at three markers (msp1, msp2 and the PolyA microsatellite) and match counting using the WHO 3/3 algorithm were used to distinguish recrudescence from new infection, PCR-uncorrected and corrected efficacy results were calculated, and molecular markers of antimalarial resistance were analyzed. Of 1777 children screened, 496 reached a study endpoint. Kaplan–Meier uncorrected efficacy for AL was 89.5% (95% CI 84–95%) in Bo, 96.1% (95% CI 92–100%) in Bombali, and 93.2% (95% CI 89–98%) in Kenema; for ASAQ, estimates were 92.0% (95% CI 86–98%) in Bo and 94.5% (95% CI 90–99%) in Bombali. PCR-corrected Kaplan–Meier efficacy was 100% in all AL arms, 100% for ASAQ in Bo, and 99.1% (95% CI 97–100%) for ASAQ in Bombali.

New infections accounted for 72.7% (16/22) and 63.6% (7/11) of recurrent parasitemia in the AL and ASAQ arms, respectively. No validated or candidate Pfk13 mutations were detected, and while some *Pfmdr1* and *Pfcrt* polymorphisms were observed, no clear pattern of association with treatment failure was evident. Therefore, our study provides evidence that AL and ASAQ remain efficacious for treatment of *P. falciparum* infection in Sierra Leone.

**Supplementary Information:**

The online version contains supplementary material available at 10.1186/s12936-026-05850-y.

## Background

In 2023, there were an estimated 263 million cases of malaria globally; 94% of cases occurred in Africa and an estimated 2.5 million cases occurred in Sierra Leone [1]. As the leading cause of  illness and death in the country, malaria poses a significant public health problem. Malaria control at the population level relies on effective case management including administration of oral artemisinin-based combination therapies (ACTs), which combine a potent short half-life artemisinin derivative with a partner antimalarial drug with a longer half-life to improve the likelihood of parasite clearance. The Sierra Leone Ministry of Health and Sanitation (MoHS) and the Sierra Leone National Malaria Control Programme (NMCP) recommend the ACT artemether-lumefantrine (AL) as first-line therapy and artesunate-amodiaquine (ASAQ) as second-line therapy for uncomplicated *Plasmodium falciparum (Pf)* infection in Sierra Leone. AL is also the most commonly used ACT in Africa and accounts for approximately 85% of all procured ACTs [2].

Resistance of *Pf* to commonly used antimalarials has traditionally represented a major obstacle to malaria treatment, particularly in Southeast Asia, where evidence for the emergence of resistance to artemisinin derivatives is well-documented [3–7]. Recently, independent emergence of artemisinin partial resistance (i.e., delayed parasite clearance following treatment) has also been reported from East Africa [8, 9]. To characterize and mitigate the risks associated with ACT resistance, the World Health Organization (WHO) recommends monitoring ACT efficacy in endemic countries using a standardized therapeutic efficacy study (TES) protocol at least every 2 years. TES are conducted to monitor the efficacy of antimalarial drugs and are the gold standard for identifying emerging resistance, guiding treatment policy updates, and ensuring that recommended therapies remain effective in controlling malaria and reducing mortality [10].

Prior to 2021, the most recent study on the therapeutic efficacy of ACTs in Sierra Leone was carried out in 2016 in three sites—Bo, Bombali, and Kenema. The TES evaluated AL, ASAQ, and dihydroartemisinin-piperaquine (DP), showing 100% PCR-corrected efficacy using the WHO’s three out of three match criteria in all sites for all drugs. For AL, uncorrected efficacy was 91.1% in Kenema. For ASAQ, uncorrected efficacy was 82% in Bo and 94% in Bombali. Uncorrected DP efficacy was 89% in Bo and 90% in Bombali [11]. The 2021–2022 TES builds on Sierra Leone’s existing body of efficacy research by re-evaluating AL and ASAQ in the same three sites evaluated in 2016, in addition to assessing for molecular markers of resistance.

## Methods

### Study design and sites

This study was conducted following the standard WHO in vivo protocol for antimalarial efficacy surveillance [10]. The evaluation was conducted between December 2021 and June 2022 in three districts of Sierra Leone: Bo District, located in southern Sierra Leone with a malaria prevalence among children aged 6–59 months of 57.1% using rapid diagnostic tests,Kenema District, located in eastern Sierra Leone with a malaria prevalence among children 6–59 months of 59.3% using rapid diagnostic tests; and Bombali District, located in northern Sierra Leone with a malaria prevalence of 47.7% among children 6–59 months using rapid diagnostic tests [12].

In two sites—Bo and Bombali—two drugs were evaluated (AL and ASAQ); one site—Kenema—evaluated only one drug (AL) due to slow enrollment as a result of the high transmission season ending. This is similar to the 2016 study, where AL and ASAQ were evaluated in Bo and Bombali Districts, and AL was evaluated in Kenema [11]. Participants with uncomplicated *Pf* infection were given one of the two antimalarials and were followed up over the course of 28 days to observe therapeutic response to the treatment.

The primary objectives of the study were to measure the clinical and parasitological efficacy of AL and ASAQ in children diagnosed with uncomplicated *Pf* malaria by determining the proportion with early treatment failure (ETF), late treatment failure (LTF) due to recrudescence, LTF due to new infection, or an adequate clinical and parasitological response (ACPR) as indicators of corrected and uncorrected efficacy. The secondary study objective was to determine the prevalence of known molecular markers of antimalarial drug resistance.

### Study population

Children between 6 months and 5 years of age seeking care at health facilities designated as evaluation sites and living within a five-kilometer radius of the evaluation health facility were considered for study enrollment. The evaluation included patients with an axillary temperature equal to or greater than 37.5 °C or a history of fever in the last 24 h, *Pf* monoinfection determined using microscopy, a parasite density between 2,000 and 200,000 P/μl, a hemoglobin level greater than or equal to 8 g/dl, and the ability to take oral medication. The study excluded patients who were malnourished, had a history of antimalarial treatment within the last two weeks, had general danger signs (e.g. inability to drink or breastfeed, persistent vomiting, convulsions, lethargy, or reduced consciousness) or severe malaria, had a mixed malaria infection, and those with a history of hypersensitivity to the study medications. The inclusion and exclusion criteria for this study are described in further detail in the 2009 WHO protocol for evaluating and monitoring the efficacy of antimalarial drugs for the treatment of uncomplicated *Plasmodium falciparum* malaria [10].

### Sample size calculation

The sample size was determined with the assumption of an expected failure rate of 5% and a loss to follow-up rate of 27%. For an evaluation attempting to measure the failure rate with a precision of 5% with a statistical power of 80%, the sample size was set at 100 participants per arm per district, totaling 200 participants each for Bo and Bombali which evaluated AL and ASAQ and 100 participants for Kenema which evaluated only AL.

### Study procedures

In all three districts, the first 100 participants were enrolled to receive AL (Coartem™ manufactured by Novartis). In Bo and Bombali, the next 100 participants received ASAQ (manufactured by Sanofi-Aventis). Drugs used in the evaluation were quality assured with lot numbers recorded and administered in accordance with manufacturer recommendations.

Subjects in the AL arm received AL twice daily with a fat-containing snack for three days. Subjects in the ASAQ arm received one dose daily for three days administered with water and no snack. Dosing of both AL and ASAQ was based on the patient’s weight. Greater detail on dosing can be found in Supplemental Table S1.

All the medications were administered at the clinic in the presence of medical personnel except for the evening doses of AL, which were administered at the patient’s home. For participants receiving AL, the local community tracer called the parent or guardian by phone at night on day 0, 1, and 2 to remind them to give the evening dose. When participants were not able to be reached by phone, a community tracer conducted a home visit. The community tracer also called one hour later to inquire about vomiting. Caregivers were given an extra dose of AL to take home with them in case the child vomited within 30 min of their evening dose. If the child vomited within 30 min, the dose was repeated immediately. If the child vomited between 30 min to 1 h after administration of the medication, half a dose was administered. Patients with persistent vomiting (more than two times in 24 h) were excluded from the evaluation and treated with parenteral therapy (injectable artesunate, injectable artemether, or quinine) and referred as necessary.

Patients who developed severe malaria or danger signs were hospitalized and treated with parenteral artesunate and excluded from the study. Rescue therapy according to national treatment guidelines was also administered in cases of early or late treatment failure.

### Patient follow-up

The parent or guardian was instructed to bring their child to all scheduled days of follow-up (day 1, 2, 3, 7, 14, 21, 28) and any time the child's clinical status worsened (unscheduled visit). In the case of worsening clinical condition, physical examination and laboratory tests were performed to determine whether the worsening was due to malaria. During each clinic visit, the participant underwent clinical examination including vital signs, general history, and evaluation of symptoms. Thick and thin Giemsa-stained blood slides were prepared and dried blood spots (DBS) consisting of capillary blood on Whatman 903 filter paper (Cytiva®) were taken at each visit except for day 1. Hemoglobin assessment was performed on days 0, 14, and 28 of the study. A complete summary of evaluations performed at each visit is summarized in Supplemental Box S1.

Participants were considered lost to follow-up if they failed to appear every day until study day 3, or for more than one day after the scheduled follow-up visit after day 3. Participants were excluded from data analysis if the parent/guardian withdrew consent, antimalarial drugs were administered outside of the study protocol, the participant had an illness during follow-up that may have interfered with correct classification of treatment outcome, there was evidence of mixed malaria infection during follow-up, or there was missing information from the patient’s record that may have led to misclassification of treatment outcome.

All participants who had recurrent parasitemia determined using microscopy were managed according to the Sierra Leone malaria treatment guidelines and followed up until their malaria infection was cleared.

### Clinical study outcomes

At the end of follow-up, response to treatment was classified according to clinical and parasitological criteria into the WHO-standardized definitions of ETF, late clinical failure (LCF), late parasitological failure (LPF), and ACPR. ETF was defined as the presence of one of the following: danger signs or severe malaria on day 1, 2 or 3 in the presence of parasitemia, parasitemia on day 2 higher than on day 0 irrespective of axillary temperature, parasitemia on day 3 with axillary temperature ≥ 37.5 °C, and parasitemia on day 3 ≥ 25% of count on day 0. LCF was defined as the presence of danger signs or severe malaria or axillary temperature ≥ 37.5 °C in the presence of parasitemia on any day between day 4 and day 28 in patients who did not previously meet any of the criteria of early treatment failure. LPF was defined as the presence of parasitemia on any day between day 7 and day 28 with axillary temperature < 37.5 °C in patients who did not previously meet any of the criteria of early treatment failure or late clinical failure. ACPR was defined as absence of parasitemia on day 28, irrespective of axillary temperature, in patients who did not previously meet any of the criteria of early treatment failure, late clinical failure or late parasitological failure (Supplemental Box S2).

### Laboratory analyses

Laboratory analyses conducted during the study (hemoglobin, thick and thin blood smears) were performed in Sierra Leone. Upon completion of patient follow-up, all molecular testing was executed in Atlanta, Georgia at the U.S. Centers for Disease Control and Prevention’s Malaria Laboratory through the Partnership for Antimalarial Resistance Monitoring in Africa, or PARMA. All laboratory work was performed in accordance with WHO recommendations.

#### Hemoglobin and microscopy

Hemoglobin was measured on day 0, 14, and 28 using portable HemoCue (AB Leo Diagnostics, Helsinborg, Sweden) machines. Thick blood smears were prepared using a 3% Giemsa solution at day 0 to determine parasitemia levels and study eligibility; parasitemia levels were also determined at each follow-up visit. Parasitemia was calculated (per microliter) as number of parasites × 8000/number of white blood cells. Thin blood smears were stained with a 10% Giemsa and read for species identification on day 0. The blood slides were read on the same day they were prepared by two different trained laboratory technicians at each study site. Slides with > 10% difference in parasite counts between the first and second technician were re-examined by a third independent microscopist who read the slide and calculated parasite density by averaging the two closest counts. Slides were considered negative if no parasites were seen after examination of 200 oil-immersion fields in a thick blood film.

#### Dry blood spots

Blood spots were collected on Whatman 903 filter paper on days 0, 2, 3, 7, 14, 21, 28 and during any unscheduled visits. If a patient was parasitemic at any day between 7 and 28, the blood sample from that day and the blood sample from day 0 (D0) were analyzed by PCR to determine the genotype of the parasites. DNA was extracted from the DBS, amplified, and sequenced. Genotypes from D0 and the day of treatment failure (DF) were compared to differentiate recrudescence from new infection. Parasites were also analyzed for the presence of molecular markers of resistance.

#### DNA extraction and photo-induced electron transfer PCR

For all patients, the DBS collected on the day of enrolment in addition to any DBS collected after day 7 in patients with recurrent parasitemia were processed in a molecular laboratory at the U.S. Centers for Disease Control and Prevention in Atlanta, GA. Four 3 mm punches were used to extract DNA from the DBS. DNA was extracted using the QIAamp DNA Mini Kit (Qiagen, Cat # 51304) according to the manufacturer’s instructions. The extracted DNA was eluted into two extractions of 100 μL each and stored at − 20 °C until use. Parasitemia was then estimated using extracted DNA through Photo-induced electron transfer PCR (PET-PCR) of all treatment failures and a randomly chosen subset of ACPRs [13].

#### Molecular correction

Genotyping was performed on DNA from patients who completed study follow up and were classified as treatment failures. Day zero (D0) and day of failure (DF) samples for patients were compared through amplification of the *msp1* and *msp2* genes and the Poly-Alpha (PolyA) microsatellite followed by capillary electrophoresis to more precisely estimate the proportion of treatment failures by distinguishing new infections from recrudescences. All markers were assessed for all samples [14]. Any difference in length of 1.5 bp or less between D0 and the DF was considered a match.

Estimates are reported based on both the WHO-recommended three-out-of-three match criteria and the two-out-of-three match criteria used for sensitivity analysis. The three-out-of-three match algorithm classifies a recurrent infection as a recrudescence only if all three genotyped markers (*msp1*, *msp2*, and PolyA) match between the initial and recurrent samples. This conservative approach prioritizes specificity, minimizing the risk of misclassifying new infections as treatment failures. The two-out-of-three match rule defines a recrudescence if any two of the three markers match, increasing sensitivity to detect true treatment failures.

In samples with recurrent infections, each allele detected at the *msp1*, *msp2*, or *PolyA* loci was treated as a distinct clone, and samples with more than one genotype at any marker were classified as polyclonal infections. The number of alleles per marker was counted for each sample, and the highest number observed across the three markers was recorded as the sample’s multiplicity of infection. Multiplicity of infection was then averaged per site.

#### Molecular markers of resistance

PCRs of all treatment failures and a subset of ACPRs were performed to amplify the full-length *Pfk13*, *Pfmdr1*, *Pfcrt*, *Pfdhfr*, *Pfdhps*, and *Pfcytb* using the Malaria Resistance Surveillance (MaRS) Next-Generation Sequencing (NGS) protocol [15]. PCRs were repeated in the case of any unsuccessful reactions and success was verified using gel electrophoresis. Generated PCR amplicons were purified using AMPure XP beads. The resulting amplicons were then processed with the Illumina DNA Prep kit, which incorporates sequential tagmentation, indexing, and intermediate purification steps (Illumina, USA).

The generated libraries were sequenced using a V2 500 cycle flow cell (Illumina) using paired end sequencing on the Illumina MiSeq next-generation sequencer. Sequences were analyzed at loci representing the major reportable single nucleotide polymorphisms (SNPs) for each sequenced gene. A sample was considered a sequencing success if at each locus there was: a percent Quality (Q) of 35 or higher, and more than 5 reads were observed. SNPs were analyzed using the Nf-NeST (https://github.com/CDCgov/Nf-NeST) toolkit using an ensemble approach of three SNP callers (samtools, gatk, and freebayes). Sequenced files are publicly available through Bioproject PRJNA428490 on NCBI.

The weighted variant allele frequency (VAF) at each polymorphic site was calculated using the formula ${\mathrm{VAF}} = \sum {_{{\text{i = 1}}}^{{\mathrm{N}}} {\mathrm{VAF}}_{{\mathrm{i}}} } {\mathrm{w}}_{{\mathrm{i}}} /\sum {{\mathrm{w}}_{{\mathrm{i}}} }$ Here, ${\mathrm{VAF}}_{\mathrm{i}}$ is the variant allele frequency for DNA sample $\mathrm{i}$, ${\mathrm{w}}_{\mathrm{i}}$ is the weight for sample $\mathrm{i}$, and $\mathrm{N}$ is the total number of individual and pooled DNA samples for each study site. For the individually sequenced samples, ${\mathrm{w}}_{\mathrm{i}}=1$.

All D0 samples and DF samples from each site were included in this analysis. DF samples annotated as a new infection were treated as a separate, unique observation and therefore contributed an additional observation to the total number of observations. The two observations (D0 and DF) in paired samples were treated as a single observation if the pair was annotated as recrudescent infection; their observed frequencies were averaged between the two samples with the average contributing an N of 1 for the averaged values. Samples with evidence of polyclonal infection were excluded from haplotype determination because alleles could not be unambiguously assigned across loci.

Haplotypes were defined only for samples consistent with single-strain infections, operationalized as those in which the major allele accounted for more than 95% of sequencing reads at all loci within a given gene. Samples not meeting the criterion were classified as polyclonal infections and were excluded from haplotype determination, as the presence of multiple parasite strains precludes unambiguous assignment of gene-level haplotypes from short read sequencing data.

### Statistical analysis

Data were analyzed using R Studio software (RStudio: Integrated Development for R. RStudio, PBC, Boston, MA). The analyses for drug efficacy were performed in two ways: (1) Kaplan–Meier survival analysis, and (2) the per-protocol method, which differ both analytic population and use of follow-up time. In the per-protocol analysis, participants with new infections or loss to follow-up were excluded entirely from the analysis, contributing neither follow-up time nor outcomes. Efficacy was estimated as the proportion of participants completing follow-up who achieved an ACPR by day 28, excluding new infections. In contrast, the Kaplan–Meier survival analysis included all enrolled participants and incorporated time-to-event information. Participants with new infections or loss to follow-up were censored at the time of the event, such that all available follow-up time prior to treatment failure or censoring contributed to the analysis.

Efficacy was expressed as treatment success over time, defined as survival (1 − cumulative risk of treatment failure) at day 28 The primary outcome is the proportion of all patients followed up to 28 days (excluding new infections) that were classified as having an adequate clinical and parasitological response. In the Kaplan–Meier survival analysis, cases of new infection and loss to follow-up are censored from the analysis. The data for these patients up until treatment failure or loss to follow-up is included in the analysis, and the primary outcome is treatment success over time, expressed as survival (1-the cumulative risk of treatment failure).

Specifically, per-protocol uncorrected efficacy was calculated by dividing ACPRs by the number with a primary outcome (i.e., ETF, LCF, LPF, and ACPR). Per protocol PCR-corrected efficacy was calculated by dividing ACPRs by the sum of ACPRs, ETFs, and the recrudescent infections; new infections were not included in the numerator or the denominator.

D0 and DF samples were further evaluated for selection of relevant *Pfcrt* mutations (M74I, N75E, K76T, A220S, Q271E, I356T, and R371I), disaggregated by treatment arm. For participants experiencing treatment failure, Jupyter Notebook’s “statsmodel” package was used to perform a McNemar’s test for mutation status between D0 and DF paired samples. The test was performed separately for ASAQ and AL treated pairs. Only discordant pairs (mutation absent at D0 and present at DF, or vice versa) contributed to the McNemar test statistic. Statistical significance was set at p < 0.05.

### Ethics

Ethical clearance for the study was obtained from the Government of Sierra Leone’s Office of the Sierra Leone Ethics and Scientific Review Committee on July 5, 2021. The study was determined to be non-engaged research by the CDC and public health surveillance by the Population Services International Research Ethics Board. Parents/guardians were informed of the study procedure, its benefits, potential risks, and gave written informed consent for their children to participate in the study prior to enrollment.

## Results

### Baseline participant characteristics

**Fig. 1:**
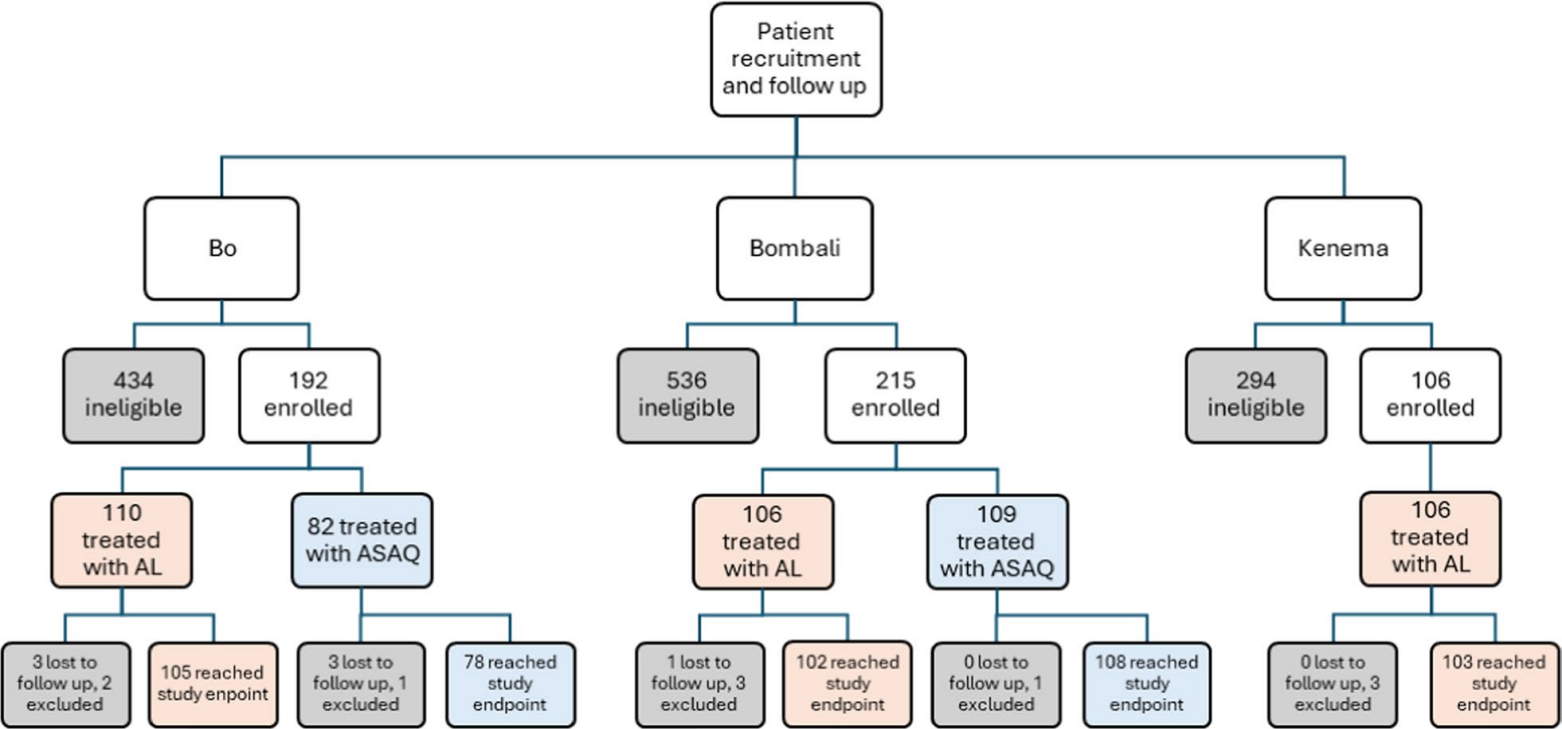
Overview of study enrollment in Sierra Leone’s 2021 therapeutic efficacy study. AL: Artemether Lumefantrine; ASAQ: Artesunate Amodiaquine

A total of 1777 children were screened in all three study sites (Fig. 1), including 1148 in the AL arm (324 in Bo, 424 Bombali, 400 Kenema) and 629 in the ASAQ arm (302 Bo, 327 in Bombali). In the AL arm 322 were enrolled in the study, with 310 (96.0%) reaching a study endpoint. In the ASAQ arm, 191 were enrolled, with 186 (97.4%) reaching a study endpoint. In each study site, patients were first given AL until the drug arm’s minimum sample size was met; upon attaining adequate AL sample size, sites transitioned to enrolling patients into ASAQ arms. The most common reasons for exclusion included mixed malaria infection and hemoglobin levels below the enrollment threshold. Additional details, including baseline participant characteristics, are shown in Table 1.

**Table 1:** Participant flow and baseline characteristics by TES study site and treatment arm in Sierra Leone, 2021

	Bo		Bombali		Kenema
	AL	ASAQ	AL	ASAQ	AL
Total screened	324	302	424	327	400
Total enrolled	110	82	106	109	106
Loss to follow up, n (%)	3 (2.7)	3 (3.7)	1 (0.9)	0 (0)	0 (0)
Exclusion, n (%)	2 (1.8)	1 (1.2)	3 (2.8)	1 (0.9)	3 (2.8)
Reached study endpoint, n (%)	105 (95)	78 (95)	102 (96)	108 (99)	103 (97)
Participant characteristics at baseline					
Median Age, n (range)	2.6 (0.6–5)	2.8 (0.5–5)	3.3 (0.5–5)	3.0 (0.6–5)	3.5 (0.6–5)
Median weight, n (range)	13 (6–19)	12 (7–17)	14 (6–22)	14 (6–24)	13 (6–20)
% Female	53%	44%	58%	48%	58%
Median baseline parasite density, n (range)	56,407 (2007–197,188)	46,563 (2567–197,813)	31,563 (2,117–198,437)	15,938 (2011–184,375)	36,670 (2038–199,688)
Baseline hemoglobin, n (range)	9.90 (8.0–13.0)	10.7 (8.0–12.1)	9.7 (8.0–12.9)	10.1 (8.0–13.4)	10.3 (8.0–13.8)

AL: Artemether lumefantrine; ASAQ: Artesunate amodiaquine

### Speciation and correlation with microscopy results

All D0 samples and DF samples from participants with recurrent parasitemia were evaluated for speciation. Of the 496 total samples, 493 samples passed pre-analytical quality check, which involved inspection for mold or contamination, confirmation of adequate blood volume and spot quality, appropriate storage conditions, and sufficient material for downstream analysis. This included 64 paired (32 pairs of D0 and DF) samples and 429 D0 samples from patients without recurrent parasitemia. DNA was extracted from 86 samples: 64 paired and 22 randomly chosen unpaired samples. Of these, 77 samples were speciated using PET-PCR, identifying 72 *Pf* mono-infections, three *Pf*/*P. malariae* co-infections, one *Pf*/*P. malariae*/*P. ovale* triple infection, and one sample with no *Plasmodium* DNA. The CT values obtained from PET-PCR were compared to the parasitemia values determined using microscopy and demonstrate strong correlation between parasitemia counting by the microscopists as well as DNA integrity as determined by PET-PCR for all three sites (Supplemental Figure S1).

### Treatment outcomes

No treatment-related severe adverse events were reported for either drug at any of the three study sites. One study participant given ASAQ in Bombali was determined to be an early treatment failure. Slide negativity rates three days after antimalarial treatment ranged from 87–95% for AL and 98–99% for ASAQ (Table 2). The proportion of each treatment outcome by study arm is shown in Table 2. Treatment failures are delineated into early and late failures, with late failures further classified as either recrudescences or new infections based on molecular genotyping.

**Table 2:** Treatment outcomes and day 3 slide negativity rates by site in Sierra Leone, 2021

	Bo		Bombali		Kenema
	AL (n = 105)	ASAQ (n = 78)	AL (n = 102)	ASAQ (n = 108)	AL (n = 103)
Day 3 slide negativity rate (95% CI)	92% (85–96)	99% (92–100)	87% (78–92)	98% (93–100)	95% (89–98)
Early treatment failures	0	0	0	1	0
Late treatment failures	11	6	4	5	7
New infections (with PCR correction and 3/3 match)	7	5	2	2	7
Recrudescences (with PCR correction and 3/3 match)	3	1	1	1	0
Indeterminate*	1	0	1	2	0
Adequate clinical and parasitological response	94 (89.5%)	72 (92.3%)	98 (96.1%)	102 (94.4%)	96 (93.2%)

Late treatment failures: late clinical failures + late parasitological failures

PCR: polymerase chain reaction

^*^Indeterminate: sample unable to be successfully processed molecularly

**Fig. 2:**
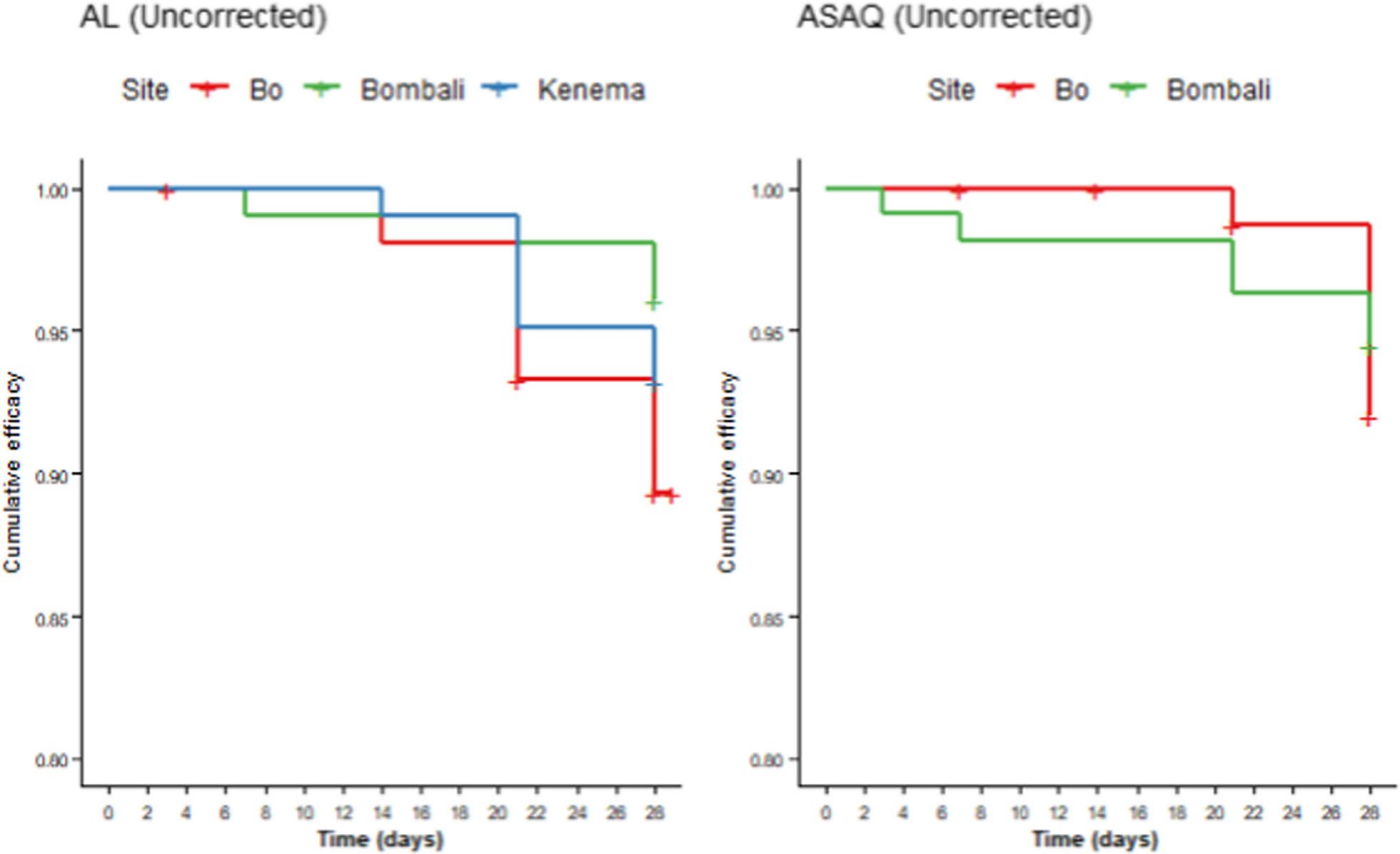
Uncorrected Kaplan–Meier efficacy of AL (left) and ASAQ (right) by study site. AL: Artemether Lumefantrine; ASAQ: Artesunate Amodiaquine

**Fig. 3:**
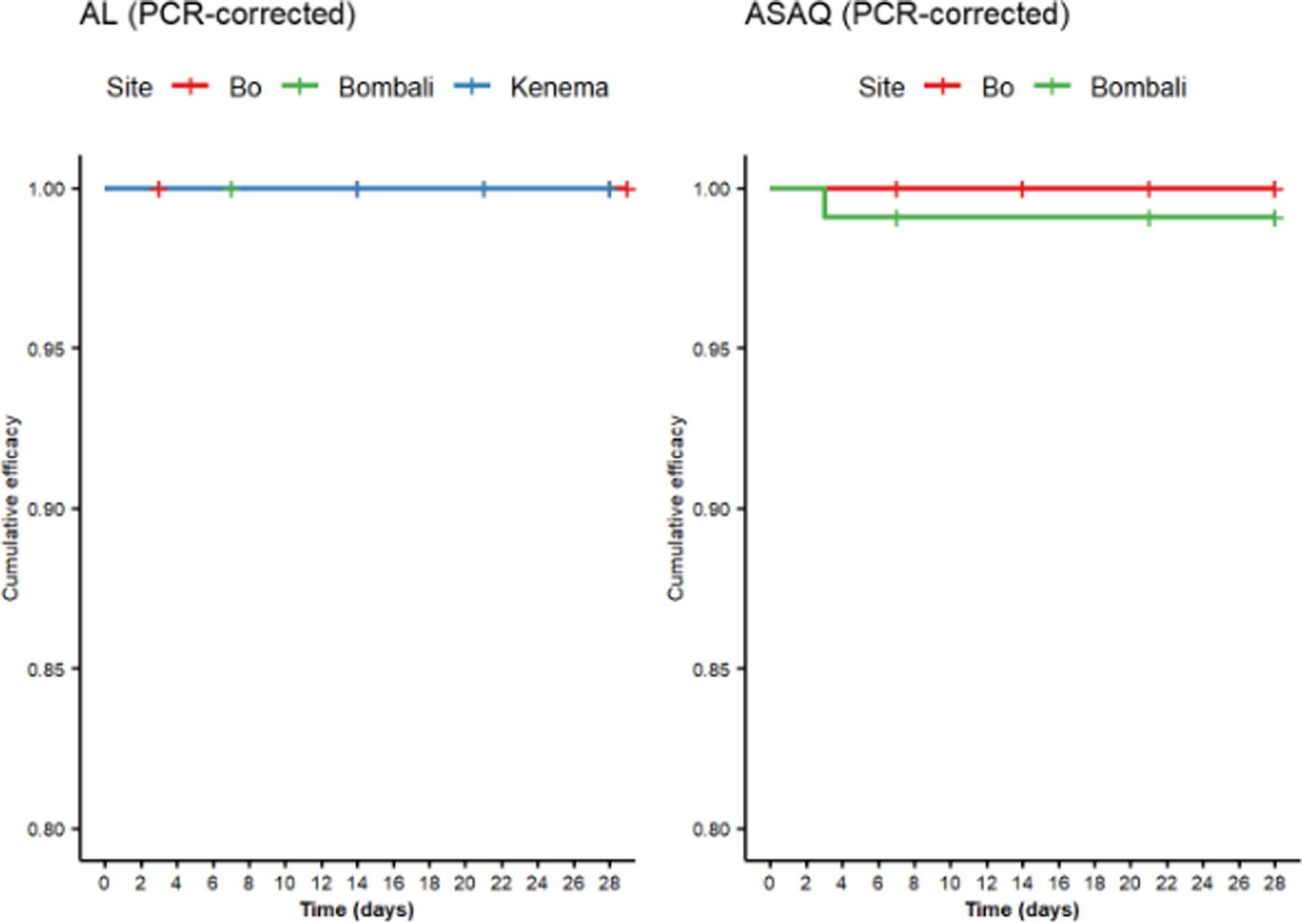
PCR-corrected Kaplan–Meier efficacy of AL (left) and ASAQ (right) by study site, WHO 3/3 match criteria. AL: Artemether Lumefantrine; ASAQ: Artesunate Amodiaquine

The results of the therapeutic efficacy of AL and ASAQ using two methods, uncorrected and PCR-corrected estimates, for both per protocol and Kaplan–Meier analyses are shown in Table 3, and Figs. 2 and 3.

**Table 3:** Treatment efficacy among participants reaching study endpoint by treatment and study site in Sierra Leone, 2021

	Bo		Bombali		Kenema
	AL (n = 105)	ASAQ (n = 78)	AL (n = 102)	ASAQ (n = 108)	AL (n = 103)
Uncorrected					
Per protocol (%) [95%CI]	89.5 [84–95]	92.3 [86–98]	96.1 [92–100]	94.4 [90–99]	93.2 [88–98]
Kaplan–Meier estimate (%) [95%CI]	89.5 [84–96]	92.0 [86–98]	96.1 [92–100]	94.5 [90–99]	93.2 [89–98]
PCR-corrected					
3/3 match–per protocol (%) [95%CI]	96.9 [93–100]	98.6 [96–100]	99.0 [97–100]	98.1 [95–100]	100*
3/3 match–Kaplan–Meier (%) [95%CI]	100*	100*	100*	99.1 [97–100]	100*
2/3 match–per protocol (%) [95%CI]	94.9 [91–99]	97.3 [94–100]	99.0 [97–100]	98.1 [95–100]	100*
2/3 match–Kaplan–Meier (%) [95%CI]	95.0 [91–99]	97.4 [94–100]	99.0 [97–100]	98.1 [96–100]	100*

AL: artemether lumefantrine; ASAQ artesunate amodiaquine; PCR: polymerase chain reaction

^*^Confidence Intervals are undefined

For AL, per protocol uncorrected efficacy was 89.5% (95% CI 84–95%) in Bo, 96.1% (95% CI 92–100%) in Bombali, and 93.2.% (95% CI 88–98%) in Kenema; these uncorrected values remained unchanged when utilizing the Kaplan–Meier approach. Using the WHO’s three-out-of-three approach, PCR-corrected per protocol efficacy was 96.9% (95% CI 93–100%) in Bo, 99.0% (95% CI 97–100%) in Bombali, and 100% in Kenema; PCR-corrected Kaplan–Meier efficacy was 100% in all three sites. Using the two-out-of-three approach, per protocol and Kaplan–Meier efficacies remained above the WHO’s 90% policy-change threshold; per protocol efficacies ranged from 94.9% (95% CI 91–99%) to 100% and Kaplan Meier Efficacies ranged from 95.0% (95% CI 91–99%) to 100%.

For ASAQ, per protocol uncorrected efficacy was 92.3% (95% CI 86–98%) in Bo and 94.4% (95% CI 90–99%) in Bombali; Kaplan–Meier uncorrected efficacy was 92.0% (95% CI 86–98%) in Bo and 94.5% (95% CI 90–99%) in Bombali. Using the WHO’s three-out-of-three approach, PCR-corrected per protocol efficacy was 98.6% (95% CI 96–100%), and 98.1% (95% CI 95–100%), respectively; using the Kaplan–Meier approach, efficacy was 100% in Bo and 99.1% (95% CI 97–100%) in Bombali. Similar to the AL arms, using the two-out-of-three approach, per protocol and Kaplan–Meier efficacies remained above the WHO’s 90% threshold; per protocol efficacies ranged from 97.3% (95% CI 94–100%) to 98.1% (95% CI 95–100%) and Kaplan Meier Efficacies ranged from 97.4% (95% CI 94–100%) to 98.1% (95% CI 96–100%). A complete genotyping database of treatment failures from all sites can be found in Supplemental Table S2.

### Assessment of molecular markers associated with antimalarial drug resistance

The presence and frequency of molecular markers associated with antimalarial drug resistance were assessed in successfully sequenced samples. Variant allele frequencies and sequencing depth for each polymorphic site in *Pfcrt*, *Pfcytb*, *Pfdhfr*, *Pfdhps*, *Pfk13*, and *Pfmdr1* are reported in Supplemental Table S3 and are visualized in Supplemental Figure S2.

None of the successfully sequenced samples analyzed for *Pfk13* had any WHO-validated or candidate *Pfk13* mutations associated with partial artemisinin resistance. For the *Pfmdr1* gene, which has been implicated in decreased sensitivity to multiple antimalarials, including lumefantrine and amodiaquine, the wild-type N86 allele predominated at this locus, with only 2.4% frequency of N86Y mutations observed in Bo, with Bombali and Kenema being 100% wild type at these nucleotide sites. The Y184F allele, putatively associated with increased resistance to lumefantrine, predominated in all sites, with 57% frequency in Bo, 60% in Bombali, and 68% in Kenema. The most common *Pfmdr1* haplotypes were the single mutant NFD (50–77%), with the wild-type NYD (23–50%) being less common.

Mutations in the *Pfcrt* gene are strongly associated with chloroquine resistance; [16, 17] additionally, certain *Pfcrt* mutations are implicated in lumefantrine and amodiaquine resistance (i.e., wild-type K76 allele has been associated with lumefantrine resistance but amodiaquine susceptibility) [18–20]. Sixteen pairs (10 ASAQ and 16 AL) of samples were successfully sequenced for the *Pfcrt* gene at the sites coding for the M74I, N75E, K76T, A220S, Q271E, I356T, and R371I mutations. In the ASAQ treated pairs, 5 pairs had at least six of the seven *Pfcrt* mutations (VAF ≥ 50%) at D0 and at DF, and 5 pairs were wild type at D0 but had the mutations at DF. In the AL treated pairs, 12 pairs were wild type at D0 and DF, 3 had mutations at D0 but not at DF, and 1 pair was wild type at D0 but had mutations at DF. McNemar’s test for within-individual changes in the presence of *Pfcrt* mutations between D0 and DF samples was not significant (p < 0.05) for either ASAQ (p = 0.0625) or AL (p = 0.625).

The triplet of canonical mutations conferring chloroquine resistance (M74I, N75E, and K76T) was found at low levels in Kenema (4.9–5%) and at moderate levels in Bo (38–40%) and Bombali (38–40%). Four other *Pfcrt* mutations (A220S, Q271E, I356T, and R371I) exhibited similar geographic patterns: mutation frequencies ranged from 4.5% to 5.4% in Kenema, 37% to 39% in Bo, and 36 to 47% in Bombali. The most common *Pfcrt* haplotypes were the wild type CVMNK (63%-95% of samples) and the triple mutant CVIET (4.8–37%).

Three *Pfdhfr* (N51I, C59R, S108N) and four *Pfdhps* (S436A, A437G, K540E, A613S) mutations associated with resistance to sulfadoxine-pyrimethamine were observed in this study. The mutant alleles N51I, C59R, and S108N were fixed (100% in all samples) in all three sites. The *Pfdhps* K540E mutation, present in the quintuple *Pfdhfr/Pfdhps* mutant, was present at low levels, ranging from 11% in Bo, 18% in Bombali, and 20% in Kenema. Furthermore, the A581G mutation, present in the sextuple *Pfdhfr/Pfdhps* mutant, was not detected in any of the three sites. Out of the 51 samples (22 from Bo, 12 from Bombali, and 17 from Kenema) that *Pfdhfr/Pfdhps* haplotypes were constructed, the quintuple haplotype IRN/ISGEAA was detected in 2 samples (9.1%, 2/22) from Bo, 2 samples (17%, 2/12) from Bombali, and 4 samples (24%, 4/17) from Kenema.

## Discussion

The results of this study provide strong evidence that both AL and ASAQ continue to perform well in Sierra Leone, with PCR-corrected efficacy above 90% observed across all sites. Uncorrected efficacy was also close to or above 90%, indicating low reinfection rates and reinforcing the drugs’ competence in preventing recurrent parasitemia during the 28-days of follow-up. After genotyping, the majority of recurrent infections were classified as new infections rather than recrudescences, indicating that both drugs remain effective in clearing initial infections. These findings are consistent with the 2016 therapeutic efficacy study, which also demonstrated high corrected efficacy for AL, ASAQ, and DP. Day 3 parasite negativity rates—87–95% for AL and 98–99% for ASAQ—were below the 90% benchmark in some AL arms. The absence of validated or candidate K13 markers in all samples is reassuring, but the increased day 3 parasitemia compared to the last study underscores the importance of ongoing monitoring for delayed clearance.

Molecular surveillance results provide additional evidence for the continued use of AL and ASAQ. No validated or candidate *Pfk13* mutations associated with artemisinin resistance were detected. While resistance-associated polymorphisms—such as *Pfmdr1* Y184F and *Pfcrt* CVIET haplotypes—were observed, these did not correspond to meaningful reductions in therapeutic efficacy. This reinforces the need to interpret molecular data alongside therapeutic outcomes, particularly in high-transmission settings where selective pressure may shape genotype frequencies without directly reducing drug efficacy.

The observed patterns of *Pfcrt* mutations provide important insights into drug-specific selection pressures. The high frequency of seven *Pfcrt* mutations (M74I, N75E, K76T, A220S, Q271E, I356T, and R371I) among ASAQ treatment failures suggests potential selection for these variants under amodiaquine pressure. However, there was no statistically significant selection for these *Pfcrt* mutations by McNemar’s test in the ASAQ pairs, nor the AL pairs. This likely reflects the small number of paired observations and the fact that McNemar’s test derives power exclusively from discordant pairs, which were limited in both treatment arms. These findings are biologically plausible given the structural similarity between amodiaquine and chloroquine, both 4-aminoquinolines, which may explain why *Pfcrt* mutations historically associated with chloroquine resistance are now being selected for under ASAQ treatment. In contrast, no such pattern was observed among AL-treated participants; the frequency of these same mutations tended to decline from D0 to DF, suggesting negative selection under lumefantrine pressure. These contrasting trends reinforce the drug-specific roles of *Pfcrt* variants and underscore the importance of continued molecular surveillance as treatment policies evolve.

Analysis of *Pfdhfr/Pfdhps* markers provided further insight into the status of SP resistance. The *Pfdhps* K540E mutation was detected at low frequencies (11–20% across sites), while the A581G mutation, a key component of the sextuple haplotype associated with high-level resistance and IPTp failure in East Africa, was absent. Among samples with constructed haplotypes, the quintuple mutant IRN/ISGEAA was observed at modest prevalence (9–24%), indicating that although resistance alleles are present, they have not reached fixation. These findings are reassuring for the continued use of SP in intermittent preventive treatment in pregnancy (IPTp) and seasonal malaria chemoprevention (SMC) in Sierra Leone, but the detection of K540E underscores the importance of ongoing surveillance to detect further expansion or emergence of highly resistant haplotypes.

The therapeutic efficacy observed in this study may also be influenced by Sierra Leone’s unique treatment history. Prior to 2015, ASAQ was widely used as the first-line treatment, with AL becoming the national first-line treatment following the Ebola-related mass drug administration campaign [21, 22]. This alternation in ACT deployment may have helped reduce pressure on AL and ASAQ, potentially contributing to their continued effectiveness. Such a pattern may also suggest that Sierra Leone could be well-positioned for the deliberate implementation of a multiple first-line therapy (MFT) strategy [23]. In addition, the country’s investment in expanding access to malaria diagnosis and treatment through its revised community health worker policy in 2021 may further support effective case management and early treatment, contributing indirectly to sustained drug efficacy [24].

## Limitations

During patient follow-up, evening doses of AL were not directly observed; although study staff placed evening reminder calls to caregivers, the lack of direct observation may affect the results of the TES. For the molecular resistance markers, while the reported genotypes and SNPs are associated with drug resistance, their predictive value for therapeutic outcomes remains limited and treatment failure can occur in the absence of these mutations. Conversely, patients can respond adequately to treatment even in the presence of these mutations. This limits the utility of molecular surveillance data alone in public health decision-making. Additionally, the samples from this study are not necessarily representative of the parasite population in the entire country, and thus molecular resistance conclusions cannot be extrapolated beyond the study sites.

Due to slow enrollment, only AL was assessed in Kenema and the ASAQ arm in Bo stopped enrolling patients before reaching the pre-determined target sample size. Furthermore, not all the samples that were collected in this therapeutic study passed quality checks for molecular work and some did not generate amplicons for sequencing. Lastly, the 407 D0 unpaired samples that were not randomly selected for sequencing were not evaluated by PET-PCR or MaRS, which limits the breadth of information gathered for these three sites.

## Conclusion

This study confirms that both AL and ASAQ remain safe and effective for the treatment of uncomplicated *P. falciparum* malaria in Sierra Leone. PCR-corrected per-protocol efficacy exceeded 96% across all study sites and treatment arms, supporting the continued use of these regimens in national policy. Molecular analysis found no WHO-validated or candidate *Pfk13* mutations associated with artemisinin resistance. Although some resistance-associated alleles were observed such as *Pfmdr1* Y184F and *Pfcrt* haplotypes (linked to lumefantrine and amodiaquine pressure) these mutations did not correspond to decreased clinical efficacy. Likewise, *Pfdhfr/Pfdhps* analysis revealed low prevalence of the quintuple haplotype and absence of the sextuple haplotype, findings that are reassuring for the continued effectiveness of SP in IPTp and SMC. Ongoing monitoring that continues to integrate both clinical outcomes and genotypic data will be critical to ensuring effective malaria case management and safeguarding antimalarial drug efficacy in Sierra Leone.

## Supplementary Information

Supplementary Material 1.

## Data Availability

The datasets used and/or analyzed during the current study will be made available upon request from the Sierra Leone Ministry of Health’s National Malaria Control Programme.

## Abbreviations

ACPR: Adequate clinical and parasitological response
ACT: Artemisinin-based combination therapy
AL: Artemether-lumefantrine
ASAQ: Artesunate-amodiaquine
D0: Day 0
DBS: Dried blood spots
DF: Day of failure
DP: Dihydroartemisinin-piperaquine
ETF: Early treatment failure
LCF: Late clinical failure
LPF: Late parasitological failure
LTF: Late treatment failure
MaRS: Malaria Resistance Surveillance
MoHS: Ministry of Health and Sanitation
MFT: Multiple first-line therapy
NGS: Next-Generation Sequencing
NMCP: National Malaria Control Programme
PET-PCR: Photo-induced electron transfer PCR
Pf: Plasmodium falciparum
SNP: Single nucleotide polymorphism
TES: Therapeutic efficacy study
WHO: World Health Organization
