# Supplementary material for: Efficacy and safety of artemether-lumefantrine (AL) and artesunate-amodiaquine (ASAQ) for the treatment of uncomplicated Plasmodium falciparum malaria among children 6–59 months in three sentinel sites of Sierra Leone, 2021–2022
Source: Malar J. 2026 Apr 9;25:182. doi: 10.1186/s12936-026-05850-y (PMC13127086; doi:10.1186/s12936-026-05850-y)
Supplement: Supplementary file 1 — Supplementary Material 1. [file 12936_2026_5850_MOESM1_ESM.docx]

**Supplemental Box S1. Summary of Evaluations for Each Visit**

| **Day** | **Clinical Exam** | **Axillary Temperature** | **Parasitemia** | **Hemoglobin** | **Filter Paper** | **Treatment** | | |
| --- | --- | --- | --- | --- | --- | --- | --- | --- |
| **Day 0** | **X** | **X** | **X** | **X** | **X** | **X** | | |
| **Day 1** | **X** | **X** | **^a^** |  | **^a^** | **X** | | |
| **Day 2** | **X** | **X** | **X** |  | **X** | **X** |  | |
| **Day 3** | **X** | **X** | **X** |  | **X** |  | | |
| **Day 7** | **X** | **X** | **X** |  | **X** |  | |  |
| **Day 14** | **X** | **X** | **X** | **X** | **X** |  | |  |
| **Day 21** | **X** | **X** | **X** |  | **X** |  | |  |
| **Day 28** | **X** | **X** | **X** | **X** | **X** |  | |  |
| **Unscheduled visit** | **X** | **X** | **X** | **X** | **X** |  | |  |
| ^a^ Parasitemia was only assessed on Day 1 if there were signs of severe malaria | | | | | | | | |

**Supplemental Box S2. Definition of Treatment Outcome Classifications**

| **Classification** | **Definition** |
| --- | --- |
| *Early Treatment Failure* | - Danger signs or severe malaria on day 1, 2, or 3 in the presence of asexual parasitemia; or - Asexual parasitemia on day 2 higher than day 0; or - Axillary temperature ≥ 37.5 °C on day 3 in the presence of asexual parasitemia; or - Asexual parasitemia on day 3 ≥ 25% of day 0 parasitemia |
| *Late Clinical Failure* | - Danger signs, signs of severe malaria, or axillary temperature ≥ 37.5 °C in the presence of parasitemia on any day between day 4 and day 28 in patients who did not previously meet any of the criteria of early treatment failure; or - Presence of asexual parasitemia on any day between day 4 and day 28 with axillary temperature ≥ 37.5 °C in patients who did not previously meet any of the criteria of early treatment failure |
| *Late Parasitological Failure* | - Presence of asexual parasitemia on any day between day 7 and day 28 with axillary temperature ≤ 37.5 °C in patients who did not previously meet any of the criteria of early treatment failure or late clinical failure |
| *Adequate Clinical and Parasitological Response* | - Absence of asexual parasitemia on day 28, irrespective of axillary temperature, in patients who did not previously meet any of the criteria of early treatment failure, late clinical failure, or late parasitological failure. |


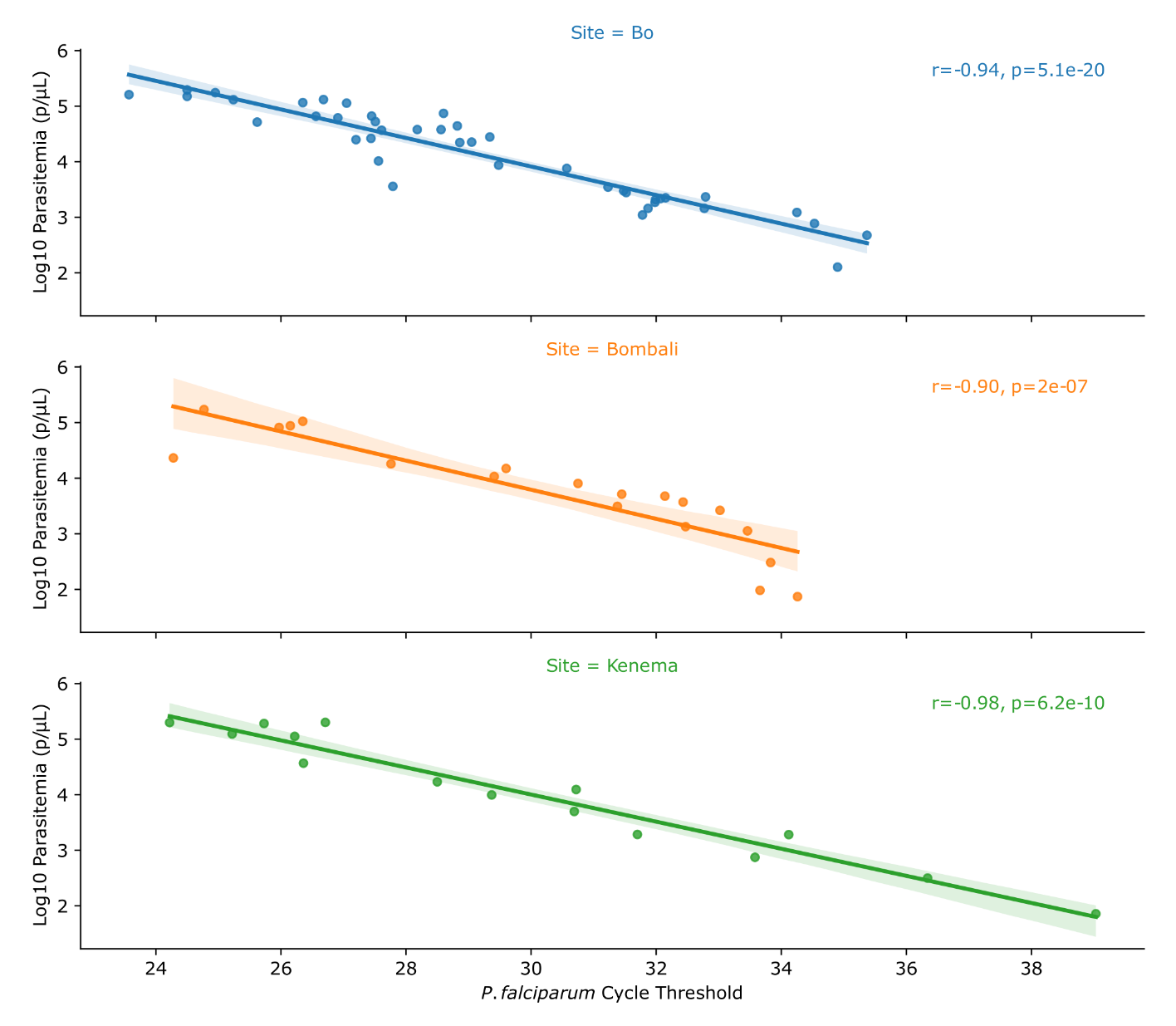


**Supplemental Figure S1. Correlation between counted parasitemia and extracted *P. falciparum* DNA quantified by PET-PCR.** Scatter plots compare the logarithmic (log10) value of the parasitemia, parasites per microliter (p/µL), (y-axis) and the cycle threshold identified using PET-PCR for the *P. falciparum* target. This is shown for all of the samples evaluated from Bo (blue), Bombali (orange**)**, and Kenema (green). The Pearson correlation (r) and p-value (p), probability of obtaining a correlation coefficient at least as extreme as the one computed from the plotted data, indicate the correlation between the data points and the line of regression for each study site plotted using Seaborn’s multiple linear regression plot (lmplot).

**Supplemental Table S1: Weight-based dosing regimens for artemether-lumefantrine and artesunate-amodiaquine**

| **Drug** | **Weight Category (kg)** | **Number of tablets** | **Dose per tablet** |
| --- | --- | --- | --- |
| artemether-lumefantrine  (AL) | 5-14.9 | 1 | 20 mg artemether / 120 mg lumefantrine |
|  | 15-24.9 | 2 |  |
|  | 25-34.9 | 3 |  |
|  | ≥ 35 | 4 |  |
| artesunate-amodiaquine  (ASAQ) | 4.5-8.9 | 1 | 25 mg artesunate / 67.5 mg amodiaquine |
|  | 9-17.9 | 1 | 50 mg artesunate / 135 mg amodiaquine |
|  | 18-35.9 | 1 | 100 mg artesunate / 270 mg amodiaquine |
|  | ≥ 36 | 2 |  |

**Supplemental Table S2. Allele-level genotyping data supporting PCR-corrected classification of treatment outcomes**

| **Sample**  **ID** | **Site** | **Drug Arm** | **K1_2** | **K1_3** | **K1_4** | **MAD20_1** | **MAD20_2** | **RO33_1** | **RO33_2** | **3D7_1** | **3D7_2** | **FC27_1** | **FC27_2** | **PolyA_1** | **PolyA_2** | **PolyA_3** | **msp1** | **msp2** | **PolyA** | **>=2/3** | **` =3/3** |
| --- | --- | --- | --- | --- | --- | --- | --- | --- | --- | --- | --- | --- | --- | --- | --- | --- | --- | --- | --- | --- | --- |
| BDL004 D0 | Bo | AL | 205.6 | 233.7 | 271.7 |  |  | 196 | 214.7 | 497.2 |  | 331.8 |  | 148.3 | 154.9 |  | **R** | **R** | **NI** | **R** | **NI** |
| BDL004 D28 | Bo | AL |  |  |  | 195.2 |  | 206.5 | 323.8 | 496.1 |  | 377.8 |  | 167.4 | 170.3 |  |  |  |  |  |  |
| BDL006 D0 | Bo | AL |  |  |  |  |  |  |  | 509.1 |  |  |  | 176.6 |  |  | **NI** | **NI** | **NI** | **NI** | **NI** |
| BDL006 D28 | Bo | AL |  |  |  |  |  |  |  | 526.9 |  | 294.5 |  | 167.4 |  |  |  |  |  |  |  |
| BDL011 D0 | Bo | AL | 243.6 |  |  |  |  | 244.1 |  | 572.7 | 590.7 |  |  | 147.2 |  |  | **R** | **IND** | **R** | **R** | **R** |
| BDL011 D28 | Bo | AL | 243.4 |  |  |  |  |  |  |  |  |  |  | 147.2 | 182.6 |  |  |  |  |  |  |
| BDL017 D0 | Bo | AL | 224.6 |  |  |  |  | 214.7 |  |  |  | 294.4 |  | 150.1 |  |  | **NI** | **NI** | **R** | **NI** | **NI** |
| BDL017 D14 | Bo | AL |  |  |  |  |  | 270.4 |  | 480 |  |  |  | 151.1 |  |  |  |  |  |  |  |
| BDL053 D0 | Bo | AL |  |  |  | 204.4 |  | 214.6 |  | 549.8 |  | 377.6 |  | 148.3 | 150.6 | 170.2 | **R** | **R** | **R** | **R** | **R** |
| BDL053 D28 | Bo | AL | 252.6 |  |  |  |  |  |  | 550.7 | 602.8 |  |  | 147.3 | 163.2 |  |  |  |  |  |  |
| BDL054 D0 | Bo | AL |  |  |  |  |  | 214.4 |  | 485.6 |  |  |  | 166.2 |  |  | **NI** | **IND** | **NI** | **NI** | **NI** |
| BDL054 D21 | Bo | AL |  |  |  |  |  |  |  |  |  |  |  | 154 |  |  |  |  |  |  |  |
| BJQ056 D0 | Bo | ASAQ | 205.2 |  |  | 241.7 |  |  |  | 458.1 |  | 331.9 |  | 154.9 |  |  | **R** | **NI** | **R** | **R** | **NI** |
| BJQ056 D28 | Bo | ASAQ |  |  |  | 103.7 | 187 | 225.2 | 243.9 | 507.6 | 517.3 | 413.8 |  | 151.7 | 154.9 |  |  |  |  |  |  |
| BJQ067 D0 | Bo | ASAQ |  |  |  |  |  | 287.2 |  | 664.6 |  | 294.5 |  | 137.1 |  |  | **NI** | **IND** | **NI** | **NI** | **NI** |
| BJQ067 D28 | Bo | ASAQ |  |  |  |  |  |  |  |  |  |  |  | 191 |  |  |  |  |  |  |  |
| BJQ073 D0 | Bo | ASAQ |  |  |  |  |  |  |  | 522.6 |  |  |  | 154.9 |  |  | **NI** | **NI** | **NI** | **NI** | **NI** |
| BJQ073 D28 | Bo | ASAQ |  |  |  | 195 |  |  |  |  |  | 377.6 |  | 169.2 |  |  |  |  |  |  |  |
| BJQ084 D0 | Bo | ASAQ |  |  |  | 195.2 |  |  |  |  |  |  |  | 167.3 |  |  | **NI** | **IND** | **NI** | **NI** | **NI** |
| BJQ084 D28 | Bo | ASAQ |  |  |  |  |  | 214.5 |  |  |  | 294.2 |  | 150.4 |  |  |  |  |  |  |  |
| BJQ094 D0 | Bo | ASAQ |  |  |  |  |  |  |  | 537.2 |  |  |  | 143.9 | 164.2 |  | **NI** | **NI** | **R** | **NI** | **NI** |
| BJQ094 D28 | Bo | ASAQ | 233.9 |  |  |  |  | 215.6 |  |  |  | 332 | 377.6 | 164.2 |  |  |  |  |  |  |  |
| BJQ100 D0 | Bo | ASAQ |  |  |  |  |  | 253.3 | 270.4 | 537.8 |  | 294.6 |  | 154 |  |  | **R** | **R** | **R** | **R** | **R** |
| BJQ100 D21 | Bo | ASAQ |  |  |  |  |  |  |  | 500.9 |  | 294.6 |  | 153.9 |  |  |  |  |  |  |  |
| BJL063 D0 | Bo | AL | 242.8 |  |  | 213.8 |  |  |  | 462.9 | 590.7 |  |  | 158.1 | 164.3 |  | **R** | **NI** | **R** | **R** | **NI** |
| BJL063 D21 | Bo | AL |  |  |  | 213.7 |  |  |  |  |  | 294.6 | 331.9 | 158.2 |  |  |  |  |  |  |  |
| BJL082 D0 | Bo | AL |  |  |  |  |  |  |  | 523.1 |  |  |  | 154 |  |  | **IND** | **IND** | **R** | **R** | **R** |
| BJL082 D14 | Bo | AL |  |  |  |  |  |  |  |  |  |  |  | 153.9 |  |  |  |  |  |  |  |
| BJL102 D0 | Bo | AL |  |  |  | 232.4 |  | 197 |  | 486.2 | 523.1 | 294.6 |  | 178.6 |  |  | **NI** | **R** | **NI** | **NI** | **NI** |
| BJL102 D21 | Bo | AL | 205.3 |  |  |  |  | 206.5 | 225.3 | 573.8 |  | 294.7 |  | 149.9 |  |  |  |  |  |  |  |
| BJL103 D0 | Bo | AL | 252.3 |  |  |  |  | 252 | 269.1 | 462.4 | 638.4 | 331.9 | 377.7 | 151.7 | 164.2 |  | **NI** | **NI** | **R** | **NI** | **NI** |
| BJL103 D21 | Bo | AL |  |  |  | 241.6 |  |  |  | 569.7 |  |  |  | 150.7 |  |  |  |  |  |  |  |
| MKL059 D0 | Bombali | AL |  |  |  | 204.3 |  | 214.6 |  | 490.6 |  | 331.9 |  | 154.8 |  |  | **R** | **NI** | **NI** | **NI** | **NI** |
| MKL059 D28 | Bombali | AL | 251.9 | 269.4 |  |  |  | 214.6 |  | 441.4 |  | 294.6 |  | 159.2 | 166 |  |  |  |  |  |  |
| MKL089 D0 | Bombali | AL |  |  |  |  |  | 214.5 |  | 532.2 |  |  |  | 172.3 |  |  | **NI** | **NI** | **NI** | **NI** | **NI** |
| MKL089 D28 | Bombali | AL | 224.5 |  |  | 204.2 |  |  |  | 549.7 | 655.5 | 413.8 |  | 161.1 | 163.2 |  |  |  |  |  |  |
| MKL105 D0 | Bombali | AL |  |  |  | 195.1 | 213.5 |  |  | 503.3 |  | 294.5 |  | 164 |  |  | **R** | **R** | **R** | **R** | **R** |
| MKL105 D7 | Bombali | AL |  |  |  | 213.6 |  |  |  |  |  | 294.5 |  | 102.8 | 163.2 |  |  |  |  |  |  |
| MDQ014 D0 | Bombali | ASAQ | 252.2 |  |  |  |  |  |  | 614.2 |  | 331.8 |  | 164.2 | 167.3 | 173.5 | **NI** | **NI** | **IND** | **NI** | **NI** |
| MDQ014 D7 | Bombali | ASAQ | 224.3 | 243.1 |  | 213.5 |  |  |  | 516.5 |  | 247.9 | 294.5 |  |  |  |  |  |  |  |  |
| MDQ034 D0 | Bombali | ASAQ |  |  |  |  |  | 214.6 |  | 577.5 |  |  |  | 131.4 |  |  | **NI** | **NI** | **NI** | **NI** | **NI** |
| MDQ034 D21 | Bombali | ASAQ |  |  |  |  |  |  |  |  |  | 294.3 |  | 157.2 |  |  |  |  |  |  |  |
| MDQ039 D0 | Bombali | ASAQ |  |  |  |  |  |  |  | 556.2 |  |  |  | 159.2 |  |  | **R** | **R** | **IND** | **R** | **R** |
| MDQ039 D28 | Bombali | ASAQ |  |  |  |  |  |  |  | 556.2 |  |  |  |  |  |  |  |  |  |  |  |
| KDL002 D0 | Kenema | AL |  |  |  | 232.3 |  | 215.9 |  | 508 | 601.5 |  |  | 151.7 |  |  | **IND** | **NI** | **NI** | **NI** | **NI** |
| KDL002 D21 | Kenema | AL |  |  |  |  |  |  |  | 482.8 |  |  |  | 164.2 |  |  |  |  |  |  |  |
| KDL007 D0 | Kenema | AL |  |  |  |  |  |  |  | 590.6 |  |  |  | 153.9 |  |  | **IND** | **NI** | **IND** | **NI** | **NI** |
| KDL007 D14 | Kenema | AL |  |  |  |  |  |  |  | 483.7 |  |  |  |  |  |  |  |  |  |  |  |
| KDL012 D0 | Kenema | AL |  |  |  |  |  | 214.7 |  | 480.5 |  |  |  | 163.1 |  |  | **R** | **IND** | **NI** | **NI** | **NI** |
| KDL012 D21 | Kenema | AL |  |  |  |  |  | 214.5 |  |  |  |  |  | 150.8 |  |  |  |  |  |  |  |
| KDL064 D0 | Kenema | AL |  |  |  | 195.1 |  |  |  |  |  | 377.6 |  | 170.4 |  |  | **NI** | **NI** | **NI** | **NI** | **NI** |
| KDL064 D28 | Kenema | AL |  |  |  |  |  |  |  | 556.1 |  |  |  | 166.2 |  |  |  |  |  |  |  |
| KDL080 D0 | Kenema | AL | 224 |  |  |  |  | 214.6 |  | 489.5 | 574.3 |  |  | 147.3 |  |  | **NI** | **NI** | **NI** | **NI** | **NI** |
| KDL080 D21 | Kenema | AL |  |  |  | 213.6 |  |  |  | 553.7 |  |  |  | 159.3 |  |  |  |  |  |  |  |
| KDL089 D0 | Kenema | AL |  |  |  | 203.9 |  |  |  | 616.4 |  |  |  | 153.5 |  |  | **NI** | **NI** | **NI** | **NI** | **NI** |
| KDL089 D28 | Kenema | AL |  |  |  | 195.1 |  |  |  | 584.2 |  |  |  | 150.7 |  |  |  |  |  |  |  |
| KDL104 D0 | Kenema | AL |  |  |  |  |  |  |  |  |  | 331.8 |  | 156.9 |  |  | **NI** | **IND** | **NI** | **NI** | **NI** |
| KDL104 D21 | Kenema | AL |  |  |  |  |  |  |  |  |  |  |  | 161.2 |  |  |  |  |  |  |  |
| NI: New Infection; R: Recrudescence; IND: Indeterminate  Bin sizes: msp1 (1.5bp); msp2 (1.5bp); polya (1.5bp) | | | | | | | | | | | | | | | | | | | | | |

**Supplemental Table S3. Mutations and haplotypes observed by site in Sierra Leone 2022 TES**

| **WHO mutation classification** | **Mutation** | **Bo** | **Bombali** | **Kenema** |
| --- | --- | --- | --- | --- |
| ***Pfk13*** | | | | |
| Validated | C580Y | 0% (n=35) | 0% (n=19) | 0% (n=22) |
|  | R539T | 0% (n=35) | 0% (n=19) | 0% (n=22) |
|  | Y493H | 0% (n=35) | 0% (n=19) | 0% (n=22) |
|  | F446I | 0% (n=35) | 0% (n=19) | 0% (n=22) |
|  | I543T | 0% (n=35) | 0% (n=19) | 0% (n=22) |
|  | P553L | 0% (n=35) | 0% (n=19) | 0% (n=22) |
|  | R561H | 0% (n=35) | 0% (n=19) | 0% (n=22) |
|  | P574L | 0% (n=35) | 0% (n=19) | 0% (n=22) |
|  | R622I | 0% (n=35) | 0% (n=19) | 0% (n=22) |
|  | A675V | 0% (n=35) | 0% (n=19) | 0% (n=22) |
| Candidate | P441L | 0% (n=35) | 0% (n=19) | 0% (n=22) |
|  | G449A | 0% (n=35) | 0% (n=19) | 0% (n=22) |
|  | C469F | 0% (n=35) | 0% (n=19) | 0% (n=22) |
|  | A481V | 0% (n=35) | 0% (n=19) | 0% (n=22) |
|  | R515K | 0% (n=35) | 0% (n=19) | 0% (n=22) |
|  | P527H | 0% (n=35) | 0% (n=19) | 0% (n=22) |
|  | N537I/D | 0% (n=35) | 0% (n=19) | 0% (n=22) |
|  | G538V | 0% (n=35) | 0% (n=19) | 0% (n=22) |
|  | V568G | 0% (n=35) | 0% (n=19) | 0% (n=22) |
| ***Pfmdr1*** | | | | |
| Candidate | N86Y | 2.4% (n=20) | 0% (n=13) | 0% (n=19) |
|  | Y184F | 57% (n=20) | 60% (n=12) | 68% (n=20) |
|  | S1034C | 0% (n=19) | 0% (n=11) | 0% (n=20) |
|  | N1042D | 0% (n=19) | 0% (n=11) | 0% (n=20) |
|  | D1246Y | 0% (n=19) | 0% (n=13) | 0% (n=19) |
| Haplotypes | NFD | 50% (n=16) | 75% (n=8) | 77% (n=13) |
|  | NYD | 50% (n=16) | 25% (n=8) | 23% (n=13) |
| ***Pfcrt*** | | | | |
| Validated | K76T | 38% (n=32) | 40% (n=18) | 5% (n=22) |
| Candidate | M74I | 38% (n=32) | 40% (n=18) | 4.9% (n=22) |
|  | N75E | 38% (n=32) | 40% (n=18) | 5% (n=22) |
|  | A220S | 38% (n=32) | 40% (n=18) | 5% (n=22) |
|  | Q271E | 39% (n=32) | 36% (n=18) | 5% (n=22) |
|  | I356T | 38% (n=32) | 41% (n=19) | 4.5% (n=22) |
|  | R371I | 37% (n=31) | 47% (n=18) | 5.4% (n=22) |
|  | N326S | 0% (n=32) | 0% (n=18) | 0% (n=22) |
|  | C72S | 0% (n=32) | 0% (n=18) | 0% (n=22) |
|  | T93S | 0% (n=32) | 0% (n=18) | 0% (n=22) |
|  | H97Y | 0% (n=32) | 0% (n=18) | 0% (n=22) |
|  | F145I | 0% (n=32) | 0% (n=18) | 0% (n=22) |
|  | I218F | 0% (n=32) | 0% (n=18) | 0% (n=22) |
|  | C350R | 0% (n=32) | 0% (n=19) | 0% (n=22) |
|  | G353V | 0% (n=32) | 0% (n=19) | 0% (n=22) |
|  | M343L | 0% (n=32) | 0% (n=19) | 0% (n=22) |
|  | V73V | 0% (n=32) | 0% (n=18) | 0% (n=22) |
| Haplotypes | CVIET | 37% (n=30) | 36% (n=14) | 5% (n=21) |
|  | CVMNK | 63% (n=30) | 64% (n=14) | 95% (n=21) |
| ***Pfdhfr*** | | | | |
| Validated | N51I | 100% (n=35) | 100% (n=19) | 100% (n=22) |
|  | C59R | 100% (n=35) | 100% (n=19) | 100% (n=22) |
|  | S108N | 100% (n=35) | 100% (n=19) | 100% (n=22) |
| Candidate | I164L | 0% (n=35) | 0% (n=19) | 0% (n=22) |
|  | A16V | 0% (n=35) | 0% (n=19) | 0% (n=22) |
| ***Pfdhps*** | | | | |
| Vaildated | A437G | 87% (n=34) | 97% (n=18) | 95% (n=22) |
|  | K540E | 11% (n=34) | 18% (n=18) | 20% (n=22) |
| Candidate | A581G | 0% (n=34) | 0% (n=18) | 0% (n=22) |
|  | I431V | 0% (n=34) | 0% (n=18) | 0% (n=22) |
| Haplotypes | IRN/IAAKAA (triple) | 14% (n=3) | 0% (n | 5.9% (n=17) |
|  | IRN/ISGKAA (quadruple) | 59% (n=13) | 75% (n=12) | 59% (n=17) |
|  | IRN/IAGKAA (quintouple) | 14% (n=3) | 0% (n=12) | 5.9% (n=17) |
|  | IRN/ISGEAA | 9.1% (n=2) | 17% (n=12) | 24% (n=17) |
|  | IRN/IAGKAS | 4.5% (n=1) | 8.3% (n=12) | 5.9% (n=17) |
| ***Pfctyb*** | | | | |
| Vaildated | Y268S | 0% (n=36) | 0% (n=19) | 0% (n=22) |
|  | Y268C | 0% (n=36) | 0% (n=19) | 0% (n=22) |
| Haplotypes | IY | 100% (n=36) | 100% (n=19) | 100% (n=22) |


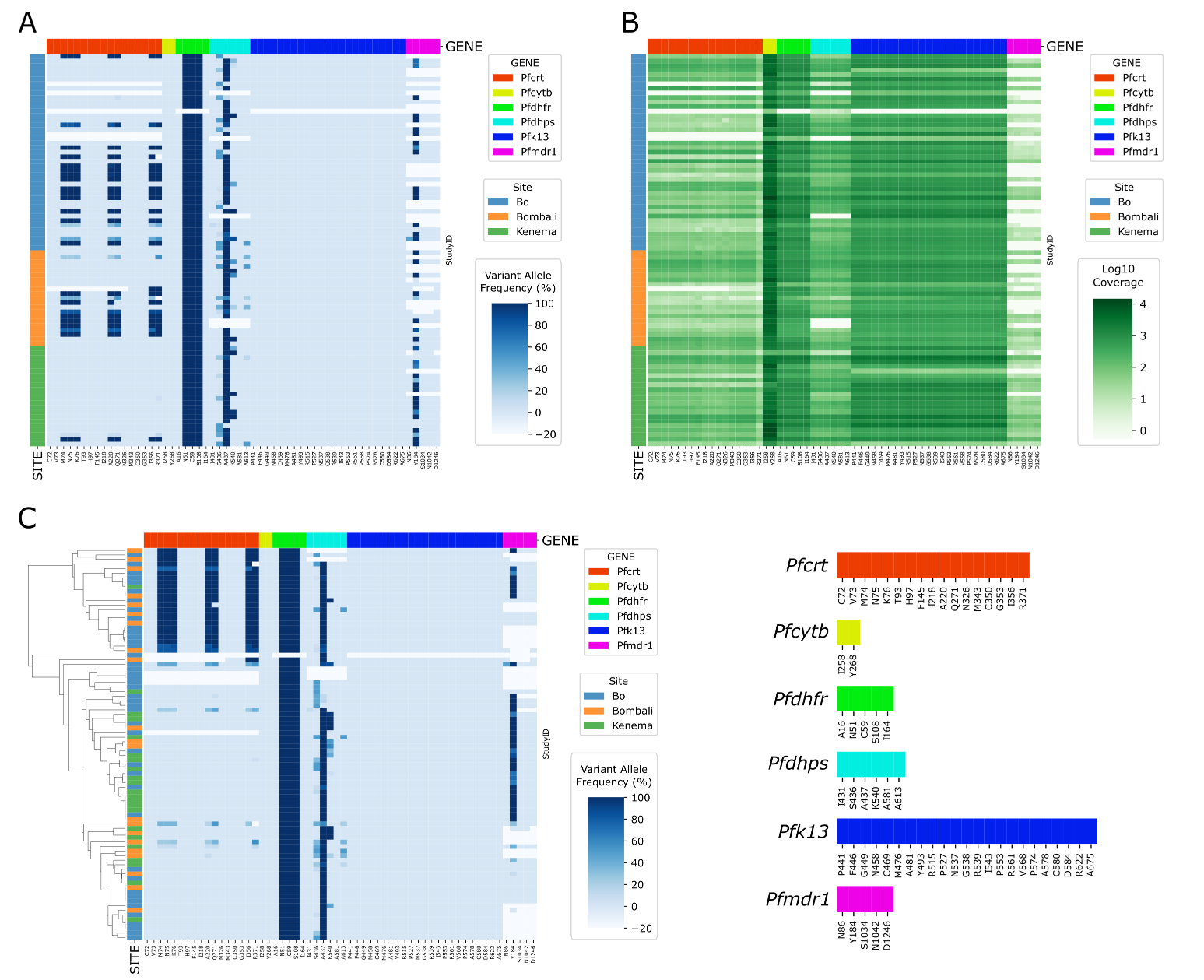

**Supplemental Figure S2. Observed mutations and sequencing coverage.** Heatmaps represent each sequenced sample (row) at each tracked amino acid for a given gene (column) for variant allele frequencies (z-axis, **A** and **C**, 0% light blue to 100% dark blue) or log10 of the number of reads (z-axis, **B**, 10^0 light green to 10^4 dark green) at each intersecting point. Each sample is labeled to the left of each heatmap with the site (blue = Bo, orange = Bombali, green = Kenema) that sample belongs to. Each visualized amino acid is denoted at the bottom of the heatmap in numerical order within the gene, color-coded at the top of the heatmap (red = *Pfcrt*, yellow = *Pfcytb*, green = *Pfdhfr*, cyan = *Pfdhps*, navy = *Pfk13*, pink = *Pfmdr1*), the amino acid belongs to. A larger listing of these amino acids per gene is shown at the bottom right of the figure. For all heatmaps, a white color (valued at -20 arbitrary units) indicates that no data was obtained for a given sample at those nucleotide positions. Samples are localized in the same rows in both of the unclustered heatmaps (**A** and **B**). **C)** Same heatmap as in **A** but the rows are hierarchically clustered based on row composition, indicating the relatedness between samples in the dataset according to the frequency of mutation(s) observed for that sample.
